# The Association of *EGFR* Mutations with Stage at Diagnosis in Lung Adenocarcinomas

**DOI:** 10.1371/journal.pone.0166821

**Published:** 2016-11-18

**Authors:** Jaeyoung Cho, Sun Mi Choi, Jinwoo Lee, Chang-Hoon Lee, Sang-Min Lee, Jae-Joon Yim, Doo Hyun Chung, Chul-Gyu Yoo, Young Whan Kim, Sung Koo Han, Young Sik Park

**Affiliations:** 1 Department of Internal Medicine, Seoul National University College of Medicine, Seoul, Republic of Korea; 2 Division of Pulmonary and Critical Care Medicine, Department of Internal Medicine, Seoul National University Hospital, Seoul, Republic of Korea; 3 Department of Pathology, Seoul National University College of Medicine, Seoul, Republic of Korea; 4 Department of Pathology, Seoul National University Hospital, Seoul, Republic of Korea; Universita degli Studi di Torino, ITALY

## Abstract

**Background:**

The prognostic role of epidermal growth factor receptor (*EGFR*) mutations in patients with lung adenocarcinomas remains controversial and the association between *EGFR* mutations and stage at the time of the initial diagnosis is debatable. In this study, we evaluated the association of *EGFR* mutations with stage at diagnosis in lung adenocarcinomas.

**Materials and Methods:**

We retrospectively analyzed 1004 consecutive patients who were diagnosed with lung adenocarcinomas and tested for *EGFR* mutations between June 2011 and December 2014.

**Results:**

*EGFR* mutations were detected in 49.2% of 1004 patients with lung adenocarcinomas. In multivariable analysis, *EGFR* mutations were significantly associated with early stage disease (stage I to II) at diagnosis (odds ratio [OR], 0.65; 95% confidence interval [CI], 0.49–0.87; *P* = 0.003). When adjusted for age, sex, smoking status, and screening, the adjusted proportion of *EGFR* mutations significantly decreased according to stage. The adjusted proportions of *EGFR* mutations were 57.6% (95% CI, 51.7%–63.3%) for stage I, 47.9% (95% CI, 36.9%–59.0%) for stage II, 47.5% (95% CI, 39.6%–55.5%) for stage III, and 43.4% (95% CI, 38.3%–48.6%) for stage IV (*P* = 0.0082).

**Conclusions:**

The presence of *EGFR* mutations is significantly associated with early stage disease at initial diagnosis in lung adenocarcinomas after adjusting for age, sex, smoking status, and screening. This finding implies that *EGFR* mutations may play a role as a positive prognostic marker.

## Introduction

The presence of epidermal growth factor receptor (*EGFR*) mutations is well known to be a predictive marker for response to tyrosine kinase inhibitors (TKIs) in advanced non-small cell lung cancer (NSCLC) [1]. However, there has been considerable debate about whether the presence of *EGFR* mutations is a favorable prognostic factor. Several studies have suggested that *EGFR* mutations may have intrinsic prognostic value in advanced NSCLC [2–7], but the role of *EGFR* mutations as a prognostic marker in resected NSCLC is uncertain [8]. Previous studies examining the prognostic value of *EGFR* mutations in postoperative patients showed that the survival benefit of *EGFR*-mutant tumors lost statistical significance in multivariable analyses after adjusting for potential confounders, although patients with *EGFR* mutations had prolonged survival compared to those with wild-type *EGFR* [9–11]. However, Izar et al [12] demonstrated that patients with *EGFR* mutations had significantly longer disease-free survival (DFS) and overall survival (OS) than patients with wild-type *EGFR* and that *EGFR* mutation status was an independent prognostic marker for DFS in completely resected stage I NSCLC. Furthermore, another recent study found that *EGFR* mutation status was an independent prognostic factor for post-recurrence survival in patients with recurrent lung adenocarcinomas following curative resection [13].

The association between *EGFR* mutations and stage at the time of the initial diagnosis has not been well explored, although stage at diagnosis is the strongest prognostic marker for survival in patients with lung cancer [14]. The previous study including patients with resected lung adenocarcinomas reported that the frequency of *EGFR* mutations was not associated with pathologic stage [15]. On the other hand, recent studies showed that *EGFR* mutations were more frequent in stage IV disease among advanced or recurrent lung adenocarcinomas [16, 17]. Because previous studies were conducted in limited populations such as patients with surgically resected or advanced lung adenocarcinomas, it has been difficult to reach comprehensive conclusions regarding the association between *EGFR* mutations and stage at diagnosis. Moreover, the previous studies were limited by the fact that they did not consider screening as a confounding factor. As low-dose computed tomography (CT) screening has been demonstrated to detect lung cancer at early stages by the National Lung Screening Trial (NLST) [18], screening should be incorporated as a confounder in studies evaluating the relationship between disease stage and driver mutations. In the present study, we retrospectively evaluated the association between *EGFR* mutations and stage at diagnosis in all stages of lung adenocarcinomas, adjusting for screening.

## Materials and Methods

### Patients and samples

Our potential study subjects included all consecutive Korean patients who were admitted with an initial diagnosis of suspected lung cancer to the Department of Internal Medicine, Seoul National University Hospital, Seoul, Republic of Korea between June 2011 and December 2014. The following inclusion criteria were used to select patients for the current study: histologically/cytologically confirmed lung adenocarcinoma and testing for *EGFR* mutations at initial diagnosis. This study was approved by the institutional review board of the Seoul National University Hospital (H-1401-033-548). The requirement for informed consent was waived.

### *EGFR* mutational analysis

Specimens for *EGFR* mutational analyses included surgically resected specimens, small biopsies, and cytology specimens of pleural effusions and sputum. In the cases of the cytology specimens, we used ethanol-fixed and paraffin-embedded cell blocks, except one case with sputum cytology. DNA was extracted from formalin-fixed, paraffin-embedded tissue as per the standard protocol. Initially, the *EGFR* mutation status of the extracted DNA was determined by nested polymerase chain reaction (PCR) followed by bidirectional direct sequencing, as previously described [9]. However, after peptide nucleic acid (PNA) clamping technology was recognized as a more sensitive method compared to direct sequencing for the detection of *EGFR* mutations in diagnostic specimens with a low proportion of tumor cells [19], PNA-mediated real-time PCR clamping replaced direct sequencing from February 1, 2013. The PNAClamp *EGFR* Mutation Detection Kit (Panagene, Inc., Daejeon, Korea) was used as previously described [19].

### Clinical and pathological variables

Clinical and pathological data collected for analysis included age at diagnosis, gender, body mass index (BMI), Eastern Cooperative Oncology Group (ECOG) performance status, smoking status, locations of primary tumors, maximal standardized uptake values (SUVs) on [^18^F]fluorodeoxyglucose positron emission tomography (FDG PET)/CT, and detection methods. Detection methods were categorized as routine medical checkups, incidental findings such as a solitary pulmonary nodule on preoperative chest radiography before the performance of surgery unrelated to lung cancer, and the presence of any symptoms related to lung cancer. The authors defined screenings as the sum of the routine medical checkups and incidental findings. Screening examinations were performed by either chest CT or radiography. The final stage was defined as pathological TNM stage for surgical cases and clinical TNM stage for nonsurgical cases at the time of initial diagnosis, according to the seventh edition of the American Joint Committee on Cancer (AJCC) Staging Manual [20]. Histologic subtypes according to the new classification of the International Association for the Study of Lung Cancer (IASLC)/American Thoracic Society (ATS)/European Respiratory Society (ERS) were analyzed in surgically resected specimens [21].

### Statistical analysis

Continuous data are presented as means ± standard deviation (SD), whereas categorical data are presented as numbers and percentages. The relationships between the clinical characteristics/*EGFR* mutations and the final stage were evaluated using the independent samples t-test for continuous variables and the χ^2^ test for categorical variables. Multivariate analyses were performed with the logistic regression model adjusting for age (as a continuous variable), sex, and variables with *P* values less than 0.1 in univariate analyses. As age is known to be associated with stage at diagnosis [22, 23] and *EGFR* mutation status [15] and *EGFR* mutations are more common in females [24], age and sex are potential confounders. The factor (ECOG performance status) showing multicollinearity was excluded from the multivariate analyses. The backward elimination method was applied to establish the final model. *P* values less than 0.05 were considered to have statistical significance. All statistical analyses were performed using Stata statistical software (Version 12.0, StataCorp LP, College Station, TX, USA).

## Results

### Patient characteristics

**Fig 1:**
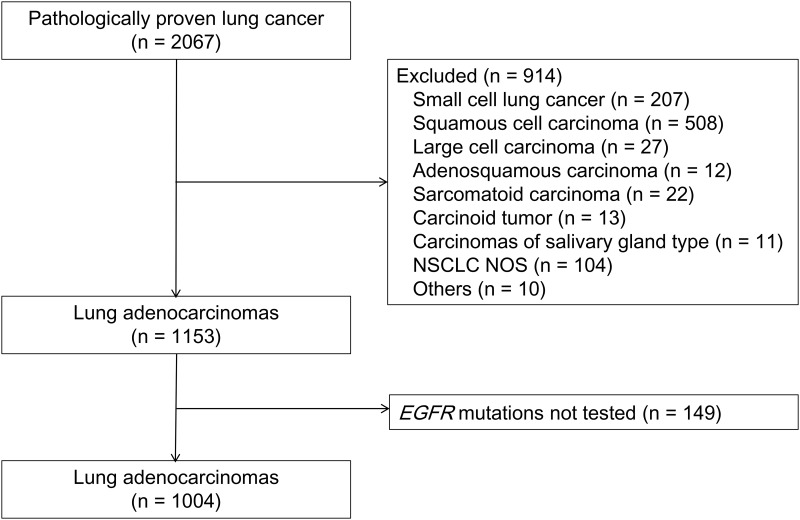
Study Population of 1004 Patients With Lung Adenocarcinomas. Abbreviations: NSCLC = non-small cell lung cancer; NOS = not otherwise specified.

Among 2067 lung cancer patients, 1153 were diagnosed with adenocarcinomas during the study period. Of these, 149 patients who did not undergo testing for *EGFR* mutations were excluded (Fig 1). The characteristics of 1004 patients with lung adenocarcinomas are listed in Table 1. The mean age of patients was 64.3 ± 10.6 years, and 522 patients (52.0%) were female. Nine hundred and fourteen patients (91.0%) had an ECOG performance status of 0 or 1 and 570 patients (56.8%) were never-smokers. Of the 1004 patients, 363 (36.2%) were diagnosed by routine medical checkups, 200 (19.9%) were diagnosed on the basis of incidental findings, and 441 (43.9%) visited the hospital with symptoms related to lung cancer. The numbers of patients in final stages I to IV were 331 (33.0%), 83 (8.3%), 161 (16.0%), and 429 (42.7%), respectively. The 1004 specimens included 504 (50.2%) resected specimens, 466 (46.4%) small biopsies, 33 (3.3%) cytology specimens of pleural effusions, and one cytology specimen of sputum. Of the 504 resected specimens, acinar adenocarcinoma was the most common histologic subtype (54.4%).

**Table 1:** Baseline Characteristics of 1004 Patients With Lung Adenocarcinomas.

Patient variables	N = 1004
Age, years, mean ± SD	64.3 ± 10.6
Female sex, n (%)	522 (52.0)
BMI, kg/m^2^, mean ± SD	23.6 ± 3.2
ECOG 0–1, n (%)	914 (91.0)
Smoking, n (%)	
Never-smoker	570 (56.8)
Ex-smoker	261 (26.0)
Current smoker	173 (17.2)
Pack-years, mean ± SD	14.2 ± 21.8
Detection methods, n (%)	
Medical checkups	363 (36.2)
Incidental findings	200 (19.9)
Any symptoms related to lung cancer	441 (43.9)
Locations (n = 997)^a^, n (%)	
RUL	287 (28.8)
RML	77 (7.7)
RLL	222 (22.3)
LUL	248 (24.9)
LLL	163 (16.4)
Maximal SUVs (n = 924)^b^, mean ± SD	10.3 ± 6.4
Final stage, n (%)	
Ia	193 (19.2)
Ib	138 (13.8)
IIa	56 (5.6)
IIb	27 (2.7)
IIIa	112 (11.2)
IIIb	49 (4.9)
IV	429 (42.7)
Types of specimens, n (%)	
Resection	504 (50.2)
Small biopsy	466 (46.4)
Cytology^c^	34 (3.4)
Histologic subtypes of resection specimens, n (%)	N = 504
Minimally invasive adenocarcinoma	6 (1.2)
Lepidic adenocarcinoma	37 (7.3)
Acinar adenocarcinoma	274 (54.4)
Papillary adenocarcinoma	69 (13.7)
Micropapillary adenocarcinoma	11 (2.2)
Solid adenocarcinoma	67 (13.3)
Invasive mucinous adenocarcinoma	35 (6.9)
Enteric adenocarcinoma	2 (0.4)
NOS	3 (0.6)

Abbreviations: SD = standard deviation; BMI = body mass index; ECOG = Eastern Cooperative Oncology Group; RUL = right upper lobe; RML = right middle lobe; RLL = right lower lobe; LUL = left upper lobe; LLL = left lower lobe; SUV = standardized uptake value; NOS = not otherwise specified.

^a^The locations of primary tumors could not be assessed in 7 cases.

^b^Eight patients did not undergo [^18^F]fluorodeoxyglucose positron emission tomography/computed tomography (FDG PET/CT). FDG PET/CT scans in 72 cases were performed at other hospitals, so we could not measure the maximal SUVs of the main masses.

^c^Cytology specimens included 33 specimens of pleural effusions and one specimen of sputum.

### Frequency of *EGFR* mutations

**Fig 2:**
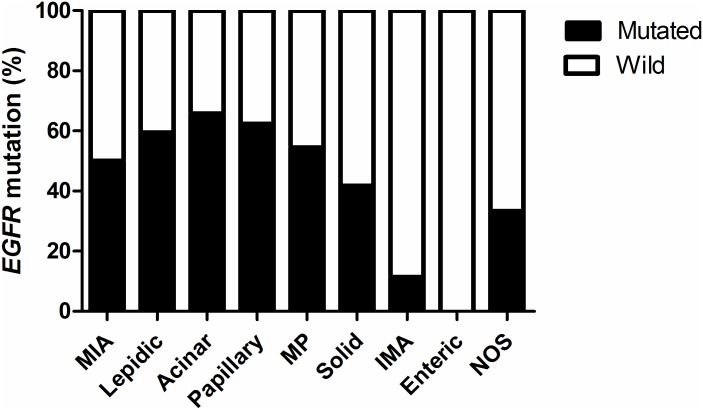
Frequencies of *EGFR* Mutations According to Histologic Subtypes. Abbreviations: MIA = minimally invasive adenocarcinoma; MP = micropapillary; IMA = invasive mucinous adenocarcinoma; NOS = not otherwise specified.

*EGFR* mutational analysis was performed by direct sequencing in 596 of the 1004 patients (59.4%) and by PNA clamping in 402 cases (40.0%). *EGFR* mutation status was unknown in 6 cases (0.6%) because mutational analysis was performed in other hospitals. *EGFR* mutations were detected in 49.2% (494 of 1004) of lung adenocarcinomas. Of the 494 *EGFR* mutations, 233 (47.2%) were deletions in exon 19; 223 (45.1%) were missense mutations in exon 21; 27 (5.5%) were mutations in exon 18; and 28 (5.7%) were mutations in exon 20. Seventeen cases had double mutations in *EGFR*. Fig 2 shows frequencies of *EGFR* mutations according to histologic subtypes. *EGFR* mutations were detected more often in acinar (65.7%), papillary (62.3%), and lepidic (59.5%) adenocarcinomas.

### Association of *EGFR* mutations with stage at diagnosis

In univariable analysis of clinical characteristics and *EGFR* mutations in relation to final stage at diagnosis, early stage disease (final stage I to II) was significantly associated with high BMI, better ECOG performance status, and never-smoking status compared to advanced stage disease (final stage III to IV) (all *P* < 0.03; Table 2). A significantly higher proportion of screened patients had early stage disease (*P* < 0.001). *EGFR* mutations were significantly correlated with early stage disease (*P* < 0.001).

**Table 2:** Univariable Analysis of Clinical Characteristics and *EGFR* Mutations for Final Stage Groups (I/II versus III/IV).

Characteristic	Early stage disease (I/II) (n = 414)	Advanced stage disease (III/IV) (n = 590)	*P*
	n (%)	n (%)	
Age, years, mean ± SD	64.8 ± 10.0	64.0 ± 10.9	0.228
Female sex	223 (53.9)	299 (50.7)	0.320
BMI, kg/m^2^, mean ± SD	23.9 ± 3.2	23.4 ± 3.1	0.007
ECOG 0–1	406 (98.1)	508 (86.1)	< 0.001
Never-smoker	252 (60.9)	318 (53.9)	0.028
Pack-years, mean ± SD	13.0 ± 21.6	15.0 ± 21.9	0.143
Screening	329 (79.5)	234 (39.7)	< 0.001
*EGFR* mutations	231 (55.8)	263 (44.6)	< 0.001

Abbreviations: SD = standard deviation; BMI = body mass index; ECOG = Eastern Cooperative Oncology Group.

**Fig 3:**
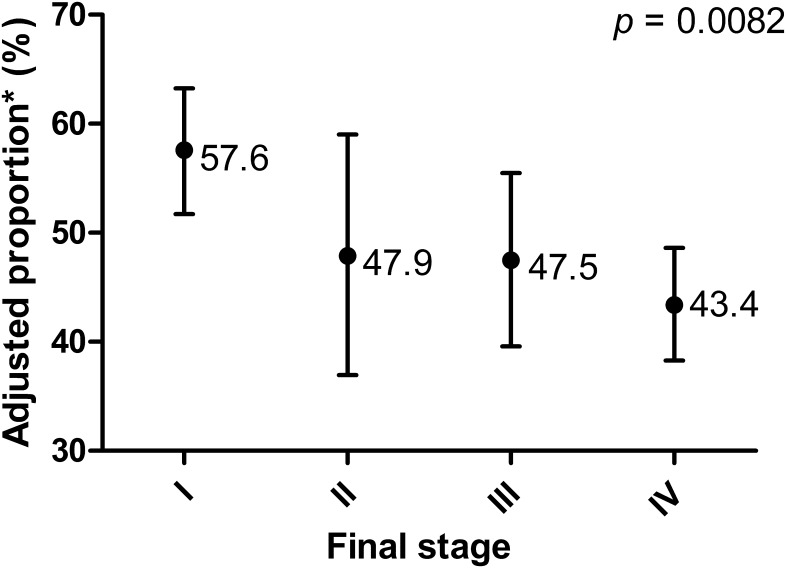
Adjusted Proportion of *EGFR* Mutations According to Final Stage. *adjusted for age, sex, smoking status, and screening.

When adjusted for age, sex, smoking status, and screening, the adjusted proportion of *EGFR* mutations significantly decreased according to final stage. The adjusted proportions of *EGFR* mutations were 57.6% (95% CI, 51.7%–63.3%) for stage I, 47.9% (95% CI, 36.9%–59.0%) for stage II, 47.5% (95% CI, 39.6%–55.5%) for stage III, and 43.4% (95% CI, 38.3%–48.6%) for stage IV (*P* = 0.0082; Fig 3). In multivariable analysis incorporating age, sex, smoking status, and screening, *EGFR* mutations were significantly associated with early stage disease (odds ratio [OR], 0.65; 95% confidence interval [CI], 0.49–0.87; *P* = 0.003; Table 3). Especially, *EGFR* mutations in exon 19 or 21 tended to be related to early stage disease (S1 Table and S1 Fig). Screening was significantly related to early stage disease (OR, 0.17; 95% CI, 0.13–0.23; *P* < 0.001) whereas age, sex, and smoking status were not associated with disease stage.

**Table 3:** Multivariable Analysis of Clinical Characteristics and *EGFR* Mutations for Final Stage Groups (I/II versus III/IV).

	OR	95% CI	*P*
Age	0.99	0.98–1.00	0.193
Female sex	1.24	0.81–1.91	0.321
Ever smoker	1.27	0.82–1.97	0.286
Screening	0.17	0.13–0.23	< 0.001
*EGFR* mutations	0.65	0.49–0.87	0.003

Abbreviations: OR = odds ratio; CI = confidence interval.

## Discussion

Findings from previous studies reporting the relationship between *EGFR* mutations and initial stage were limited to selected populations such as patients with surgically resected or advanced lung adenocarcinomas, and screening was not considered as a confounding factor [15–17]. To our knowledge, this study is the first large-scale, comprehensive analysis evaluating the association between *EGFR* mutations and stage at diagnosis in lung adenocarcinomas from an unselected population. We recruited 1004 consecutive patients with lung adenocarcinomas regardless of initial stage and treatments, and tumor samples such as biopsy, surgical, or cytologic specimens were included if sufficient tissue were available for *EGFR* mutational analysis. In our study, the frequency of *EGFR* mutations was 49.2%. We demonstrated that *EGFR* mutations were significantly associated with early stage disease after adjusting for age, sex, smoking status, and screening. This finding suggests that *EGFR* mutations may play a role as a prognostic marker.

The prognostic role of *EGFR* mutations in advanced NSCLC was suggested by retrospective analysis of the BR.21 trial, a randomized phase III trial of EGFR TKI monotherapy as second- or third-line treatment in patients with NSCLC. In the placebo arm of the BR.21 trial, patients with *EGFR* exon 19 deletions or exon 21 Leu858Arg point mutations had longer median survival than patients with wild-type *EGFR* (9.1 vs. 3.5 months, respectively) [2, 3]. Consistent results were reported from both a phase III TRIBUTE trial and a phase III INTACT trial comparing chemotherapy to chemotherapy plus EGFR TKIs in previously untreated advanced NSCLC, in which *EGFR* mutations were associated with longer survival, regardless of whether patients received EGFR TKIs [4, 5].

The prognostic value of *EGFR* mutations in resected NSCLC is unclear [8–12, 25, 26]. Several studies found that the prognostic advantages of *EGFR*-mutant tumors were lost in multivariable analyses after adjusting for potential confounders [9–11]. Kim et al [9] analyzed a series of 863 Korean patients with NSCLC who underwent surgical resection. They found that patients with *EGFR* mutations had a longer survival time compared to those with wild-type *EGFR* (*P* = 0.001). However, in multivariable analysis, *EGFR* mutation status was no longer an independent factor. The authors explained that this was because *EGFR* mutation status was not a true prognostic factor but was frequently associated with other favorable prognostic factors. This study was limited by a short mean follow-up duration of 23.6 months. Moreover, the administration of systemic chemotherapy, radiation, or EGFR TKIs affected the interpretation of the prognostic value of *EGFR* mutations on survival. A recent study by Izar et al [12] enrolled 307 patients with completely resected stage I NSCLC who received neither adjuvant nor neoadjuvant therapy, including EGFR TKIs. The results showed that patients with *EGFR* mutations had a lower rate of recurrence (9.7% vs. 21.6%; *P* = 0.03) and both longer median DFS (8.8 vs. 7.0 years; *P* < 0.01) and improved 5-year OS (98% vs. 73%; *P* = 0.003) compared to patients with wild-type *EGFR*. *EGFR* mutation status was an independent prognostic marker for DFS in a Cox regression model (hazard ratio [HR], 0.33; 95% CI, 0.12–0.87; *P* = 0.03).

The relationship between *EGFR* mutations and stage at initial diagnosis has not been well evaluated. Zhang et al [15] performed a retrospective study to evaluate the correlation between driver mutations and clinicopathological features in 349 resected lung adenocarcinomas from female never-smokers. In the study, the frequency of *EGFR* mutations was not associated with early stage disease (stage I to II) after adjusting for age, tumor differentiation, and histological subtype (OR, 1.46; 95% CI, 0.84–2.54; *P* = 0.18). Conflicting results were reported by Usuda et al [27] in a cohort of 148 Japanese patients with operable lung cancer. The clinical stage of *EGFR* mutant tumors was significantly earlier than that of wild-type tumors (*P* = 0.016). However, the study population was relatively small and heterogeneous, including 5 small cell carcinomas. On the other hand, a prospective epidemiological study of *EGFR* mutations in patients from Asia with newly diagnosed stage IIIB/IV lung adenocarcinomas showed that the frequency of *EGFR* mutations was significantly higher among patients with stage IV compared with IIIB disease (53.5% vs. 43.2%; *P* = 0.009) [16]. Similarly, Sholl et al [17] reported that *EGFR* mutations were significantly associated with stage IV disease compared with stage I to III at diagnosis in 1007 patients with stage IV or recurrent lung adenocarcinomas (26% vs. 18%; *P* = 0.004). However, the previous studies were conducted in limited populations. Furthermore, in these studies, screening status influenced the relationship of *EGFR* mutations with stage at diagnosis. In the present study, we did show that *EGFR* mutations were significantly associated with early stage disease at diagnosis after adjusting for screening in a cohort of 1004 patients with lung adenocarcinomas.

Our findings suggest that *EGFR* mutant tumors have more indolent biology. This is supported by a previous investigation of the volume doubling time (VDT) in incidentally detected symptom-free NSCLC. In the study, the VDT in the 33 adenocarcinoma patients with *EGFR* mutations was longer than that in 36 adenocarcinoma patients with wild-type *EGFR* (676 vs. 200 days; *P* = 0.014) [28]. Several studies have suggested that lung cancer with a VDT of more than 400 days indicates an indolent tumor [29, 30]. *EGFR* mutations are significantly associated with the low-grade group (adenocarcinoma in situ [AIS]/minimally invasive adenocarcinoma [MIA]) and the intermediate-group (lepidic/acinar/papillary/invasive mucinous adenocarcinoma) rather than the high-grade group (solid/micropapillary). The IASLC/ATS/ERS classification has been demonstrated to predict survival, and the low- and intermediate-grade groups have shown better survival compared with the high-grade group [31–33]. These findings suggest that *EGFR* mutations may have a role in influencing better prognosis in lung adenocarcinomas.

Our study has several limitations, such as its retrospective design of a cohort at a single institution. However, when we performed a similar analysis in 1002 patients with lung adenocarcinomas in the same cohort who were tested for anaplastic lymphoma kinase (*ALK*) rearrangements, *ALK* rearrangements were detected in 6.4% (64 of 1002) of lung adenocarcinomas and *ALK* rearrangements were significantly associated with advanced stage disease (OR, 3.78; 95% CI, 1.92–7.43; *P* < 0.001) in multivariable analysis incorporating age, sex, smoking status, and screening (S2 Table and S2 Fig), findings consistent with previous studies [34, 35]. Another limitation of this study was a higher proportion of direct sequencing of *EGFR* in advanced stage disease. As available tumor samples for mutational analyses in advanced stage cancers were limited to small biopsies (378 [88.1%] of 429 specimens of stage IV lung adenocarcinomas) or cell blocks of cytologic specimens (33 [7.7%] of 429), detection of *EGFR* mutations by direct sequencing might be suboptimal in advanced stage disease. In this study, direct sequencing was performed in 243 (56.6%) of 429. When considering intratumoral heterogeneity of *EGFR* mutations, the samples might not be representative of the whole tumors, especially in advanced stage disease. In addition, survival data are lacking. Because the data were collected from 2011 to 2014, a longer follow-up time will be required to obtain reliable survival data.

## Conclusion

In conclusion, we found that the presence of *EGFR* mutations was significantly associated with early stage disease at initial diagnosis in lung adenocarcinomas. The data suggest that *EGFR* mutations may have a prognostic role and *EGFR* mutant tumors seem to be more indolent.

## Supporting Information

S1 Fig Adjusted Proportion of *EGFR* Mutations in Exon 19 or 21 According to Final Stage. *adjusted for age, sex, smoking status, and screening.
(TIF)

S2 Fig Adjusted Proportion of *ALK* Rearrangements According to Final Stage. *adjusted for age, sex, smoking status, and screening.
(TIF)

S1 Table Multivariable Analysis of Clinical Characteristics and *EGFR* Mutations in Exon 19 or 21 for Final Stage Groups (I/II versus III/IV). (DOCX)

S2 Table Multivariable Analysis of Clinical Characteristics and *ALK* Rearrangements for Final Stage Groups (I/II versus III/IV). (DOCX)
