# Supplementary figures and images for: The Association of EGFR Mutations with Stage at Diagnosis in Lung Adenocarcinomas
Source: PLoS One. 2016 Nov 18;11(11):e0166821. doi: 10.1371/journal.pone.0166821 (PMC5115811; doi:10.1371/journal.pone.0166821)

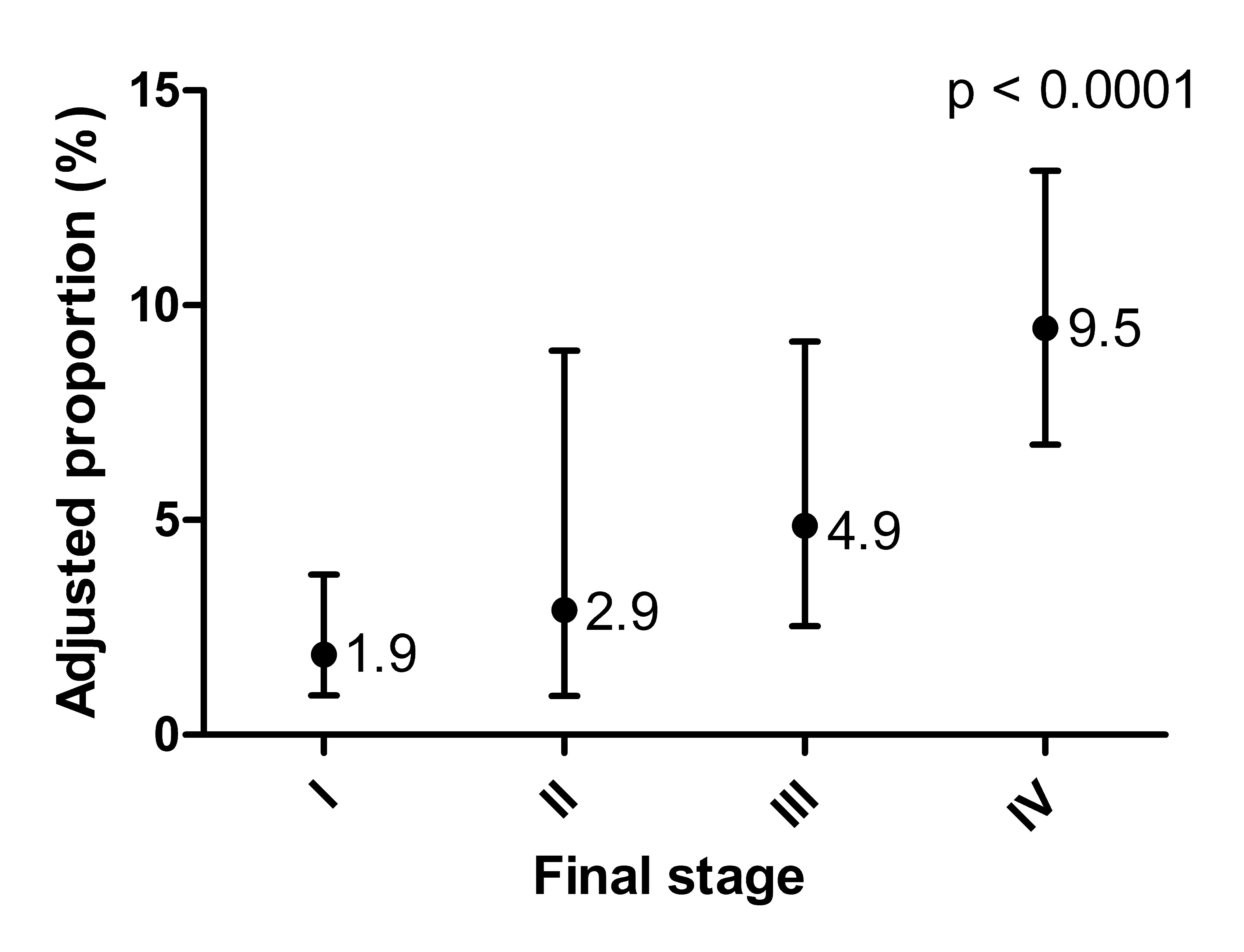

Supplement: S2 Fig — *adjusted for age, sex, smoking status, and screening. (TIF) [file pone.0166821.s002.tif]
