# Supplementary material for: The Association of EGFR Mutations with Stage at Diagnosis in Lung Adenocarcinomas
Source: PLoS One. 2016 Nov 18;11(11):e0166821. doi: 10.1371/journal.pone.0166821 (PMC5115811; doi:10.1371/journal.pone.0166821)
Supplement: S1 Table — (DOCX) [file pone.0166821.s003.docx]

**Supplemental Table 1**

Multivariable Analysis of Clinical Characteristics and *EGFR* Mutations in Exon 19 or 21 for Final Stage Groups (I/II versus III/IV)

|  | OR | 95% CI | *P* |
| --- | --- | --- | --- |
| Age | 0.99 | 0.98–1.00 | 0.187 |
| Female sex | 1.26 | 0.82–1.94 | 0.293 |
| Ever smoker | 1.28 | 0.83–1.98 | 0.271 |
| Screening | 0.17 | 0.13–0.23 | < 0.001 |
| *EGFR* mutations in exon 19 or 21 | 0.64 | 0.48–0.85 | 0.002 |

Abbreviations: OR = odds ratio; CI = confidence interval
