# Supplementary material for: The Association of EGFR Mutations with Stage at Diagnosis in Lung Adenocarcinomas
Source: PLoS One. 2016 Nov 18;11(11):e0166821. doi: 10.1371/journal.pone.0166821 (PMC5115811; doi:10.1371/journal.pone.0166821)
Supplement: S2 Table — (DOCX) [file pone.0166821.s004.docx]

**Supplemental Table 2**

Multivariable Analysis of Clinical Characteristics and *ALK* Rearrangements for Final Stage Groups (I/II versus III/IV)

|  | OR | 95% CI | *P* |
| --- | --- | --- | --- |
| Age | 1.00 | 0.98–1.01 | 0.472 |
| Female sex | 1.17 | 0.76–1.81 | 0.476 |
| Ever smoker | 1.45 | 0.93–2.25 | 0.099 |
| Screening | 0.17 | 0.13–0.23 | < 0.001 |
| *ALK* rearrangements | 3.78 | 1.92–7.43 | < 0.001 |

Abbreviations: OR = odds ratio; CI = confidence interval
